# Progressive structural changes of Avicel, bleached softwood, and bacterial cellulose during enzymatic hydrolysis

**DOI:** 10.1038/srep15102

**Published:** 2015-10-14

**Authors:** Kabindra Kafle, Heenae Shin, Christopher M. Lee, Sunkyu Park, Seong H. Kim

**Affiliations:** 1 Department of Chemical Engineering and Material Research Institute, The Pennsylvania State University, University Park, PA 16802, USA; 2 Department of Forest Biomaterials, North Carolina State University, Raleigh, NC 27695, USA

## Abstract

A comprehensive picture of structural changes of cellulosic biomass during enzymatic hydrolysis is essential for a better understanding of enzymatic actions and development of more efficient enzymes. In this study, a suite of analytical techniques including sum frequency generation (SFG) spectroscopy, infrared (IR) spectroscopy, x-ray diffraction (XRD), and x-ray photoelectron spectroscopy (XPS) were employed for lignin-free model biomass samples—Avicel, bleached softwood, and bacterial cellulose—to find correlations between the decrease in hydrolysis rate over time and the structural or chemical changes of biomass during the hydrolysis reaction. The results showed that the decrease in hydrolysis rate over time appears to correlate with the irreversible deposition of non-cellulosic species (either reaction side products or denatured enzymes, or both) on the cellulosic substrate surface. The crystallinity, degree of polymerization, and meso-scale packing of cellulose do not seem to positively correlate with the decrease in hydrolysis rate observed for all three substrates tested in this study. It was also found that the cellulose Iα component of the bacterial cellulose is preferentially hydrolyzed by the enzyme than the cellulose Iβ component.

Lignocellulosic biomass has recently gained significant interests as a renewable energy source. For example, ethanol can be produced by fermentation of glucose obtained from enzymatic hydrolysis of cellulosic biomass^1^. The hydrolysis rate of cellulosic biomass by enzymes is initially high and then gradually decreases over time. The factors involved in this behavior have been deemed to arise from denaturation of enzymes as well as changes in the overall crystallinity (*i.e*., amorphous versus crystalline) or polymorphic ratio (if more than one type of cellulose is present) of cellulosic substrates during the hydrolysis, cellulose crystal size and/or degree of polymerization, and cellulose accessibility for enzymes^2^,^3^. The accessibility can be further divided into two categories: the transport of enzymes through the substrate matrix to the cellulose and the exposure of the enzyme binding sites at the cellulose surface. Changes in any of these traits could affect the hydrolysis enzymatic activity.

It is known that enzymes hydrolyze amorphous cellulose faster than crystalline cellulose^2^,^3^. Previous studies reported a positive correlation between hydrolysis rate and crystallinity index for isolated cellulose samples^4^,^5^,^6^. However, the opposite was also reported when real lignocellulose samples were used for hydrolysis^7^. In addition to crystallinity, changes in degree of polymerization (DP), or length of cellulose chains, were also considered as a factor for the decreased rate of enzymatic hydrolysis^3^,^8^.

In order to improve the accessibility of enzymes to the cellulose substrate, lignocellulose biomass is typically pretreated via various methods such as dilute acid, autohydrolysis, alkaline, or steam explosion pretreatment^9^,^10^. However, aggregation of cellulose microfibrils could occur after removal of lignin and matrix polymers during pretreatments^11^,^12^,^13^. The aggregation of cellulose microfibrils might negatively affect the surface accessibility^14^,^15^.

In this study, three lignin-free model cellulosic substrates (Avicel, bleached softwood, and bacterial cellulose) were analyzed to monitor changes in crystallinity, crystal size, chain length, meso-scale packing, and surface contamination of substrates over the course of enzymatic hydrolysis. Although their compositions and physical structures were different, all three substrates showed a similar behavior in hydrolysis rate reduction during the enzymatic reaction. Therefore, it is hypothesized that there must exist common features or changes that could be correlated with such decreases in hydrolysis rate. This study uses a suite of analytical techniques covering various structural aspects of cellulose at multiple length scales^16^. X-ray diffraction (XRD) was employed to analyze the cellulose structure and determine the cellulose crystallinity and crystal size of cellulosic samples^17^,^18^,^19^. Infrared (IR) spectroscopy was used to check the polymorphic structures of cellulose (cellulose Iα, Iβ, II, III)^20^,^21^. X-ray photoelectron spectroscopy (XPS) was utilized to monitor changes in the surface composition of cellulosic substrates^22^. Vibrational sum-frequency-generation (SFG) spectroscopy was used to nondestructively probe crystal structure of cellulose as well as meso-scale packing of crystalline cellulose domains inside biomass 18,20,23,24,25,26,27,28. The comparison of structural changes found by each analytical method, with temporal changes in enzymatic hydrolysis yield, revealed the dominant factor common to all three substrates.

## Materials and Methods

### Cellulose substrates

Avicel, a highly-crystalline pure cellulose Iβ substrate, was commercially purchased. Bleached softwood pulps were obtained from a local mill near North Carolina State University. The bleached softwood consisted of ~80% cellulose and ~20% hemicellulose. Bacterial cellulose was prepared as described in a previous paper^29^.

### Enzymatic hydrolysis

Cellulase (NS 50013), xylanase (NS50014), and β-glucosidase (NS 50010) were received from Novozymes (Franklinton, NC) and used as a mixture at a ratio of 1:0.3:0.3 by weight. An enzyme dosage of 5 FPU/g substrate was used for bleached softwood, 10 FPU/g substrate for bacterial cellulose, and 20 FPU/g substrate for Avicel. The enzyme loading for each substrate was adjusted so that similar levels of carbohydrate conversion were obtained for the three model biomass samples. All enzymatic hydrolysis were performed in 100 mL of 100 mM acetate buffer (pH 5.0) at a 5% (w/v) solid loading. The substrate and enzyme mixtures were incubated in a shaking water bath at 50 °C and 150 rpm. The hydrolysis yield was measured after reactions for 12, 24, 48, 72, and 96 hours. All samples retrieved from the reaction vessel were thoroughly washed with deionized water, freeze-dried, and stored for further analytical analysis.

### Compositional analysis

The sugar and lignin contents of the sample were determined according to the standard procedure suggested by the National Renewable Energy Laboratory (NREL)^30^. The filtrate obtained from acid digestion was analyzed with high-performance liquid chromatography (Dionex, Sunnyvale, CA) to quantify the amount of structural sugars. Sugars were separated through a Shodex SP0810 column at a temperature of 80 °C. The compositions of Avicel and bleached softwood determined in this method are presented in Table 1.

### Degree of polymerization (DP)

The DP of cellulose was measured by gel permeation chromatography (GPC) after the carbanilation treatment^31^. Briefly, a 25 mg sample of dry biomass was placed in a small glass vial with 10 mL of anhydrous pyridine and 1 mL of phenyl isocyanate was added slowly to the vial. The vial was gently stirred for 48 hours for carbanilation. Methanol was added to consume any remaining phenyl isocyanate. The clear solution was poured into a 70:30 methanol/water mixture to precipitate carbanilated cellulose. The solid was collected and washed three times with methanol. After freeze drying, it was dissolved in tetrahydrofuran and injected in GPC system. Polystyrene standards were used to make a calibration curve. The apparent molecular weight was divided by 519 (the molecular weight of repeating unit of carbanilated cellulose with 3 degrees of substitution) to calculate the weight average degree of polymerization (DP_w_).

### X-ray diffraction (XRD)

XRD experiments were performed using a Rigaku SmartLab x-ray diffraction with a Cu tube (λ = 1.5405 Å). The diffraction angle of 2θ from 9° to 41° was measured at a step size of 0.05°. Cellulose crystallinity was calculated based on the peak height method^32^. Crystal size was calculated using Scherrer’s equation from the (200) peak width for Avicel and bleached softwood, and the (110) peak width for bacterial cellulose^19^.

### Vibrational sum frequency generation (SFG) spectroscopy

Pellets were made from the powdered samples retrieved at various hydrolysis time points. The instrumental details of SFG have been described elsewhere^25^. SFG spectra were taken at a 4 cm^−1^ interval in the CH stretch vibration region (2,700–3,050 cm^−1^) and an 8 cm^−1^ interval in the OH stretch vibration region (3,100–3,800 cm^−1^). Each data point was an average of 100 laser shots, and the measured SFG signal intensity was normalized with the IR and visible input laser intensities at each shot. Each spectrum reported here represents an average of 10 different locations of the sample pellet. The SFG probe volume has been estimated to be 200 μm × 150 μm on the surface and up to 20 μm deep from the external surface^26^. For polymorphic analysis, SFG spectra of bacterial cellulose were deconvoluted using a Lorentzian function fit^23^. The hydroxyl region was deconvoluted with five components, and the cellulose Iα/(Iα + Iβ) ratio was calculated as A_3240_/(A_3240_ + A_3270_) where A_3240_ and A_3270_ are peak areas of the ~3,240 cm^−1^ and ~3,270 cm^−1^ peaks^23^.

### X-ray photoelectron spectroscopy (XPS)

XPS analysis was performed using a Kratos Axis Ultra, equipped with a monochromatic Al Kα radiation. Pristine samples, as well as samples retrieved after 12 h hydrolysis and the final hydrolysis residue (96 h for Avicel and bleached softwood and 72 h for bacterial cellulose) were analyzed. All spectra were acquired from a sample area of 1 mm × 1 mm.

### Attenuated total reflectance infrared (ATR-IR) spectroscopy

ATR-IR spectra were collected using a Nicolet 8700 Research FT-IR Spectrometer (Thermo Scientific) equipped with a smart iTR diamond ATR unit, a KBr beam splitter, and a deuterated triglycine sulfate detector. All spectra were collected from the pressed sample pellet in the region of 650–4,000 cm^−1^ with a 4 cm^−1^ resolution and averaged over 100 scans. All the spectra were normalized to ~1,031 cm^−1^ for representation purposes.

## Results and Discussion

The experimental results are discussed in the following order. First, the enzymatic hydrolysis yield, as a function of reaction time, is described in Section 1. Then, it is discussed if the decrease in enzymatic hydrolysis rate of cellulose can be correlated with changes in cellulose chain length from DP measurements (Section 2), crystallinity, and crystal size from XRD (Section 3), meso-scale arrangement of cellulose microfibrils from SFG (Section 4), and surface contamination from XPS (Section 5). Finally, the polymorphic structure change of bacterial cellulose during enzymatic hydrolysis is discussed from SFG, ATR-IR, and XRD analyses (Section 6).

### Common characteristics in enzymatic hydrolysis—decrease in reaction rate over time

**Figure 1:**
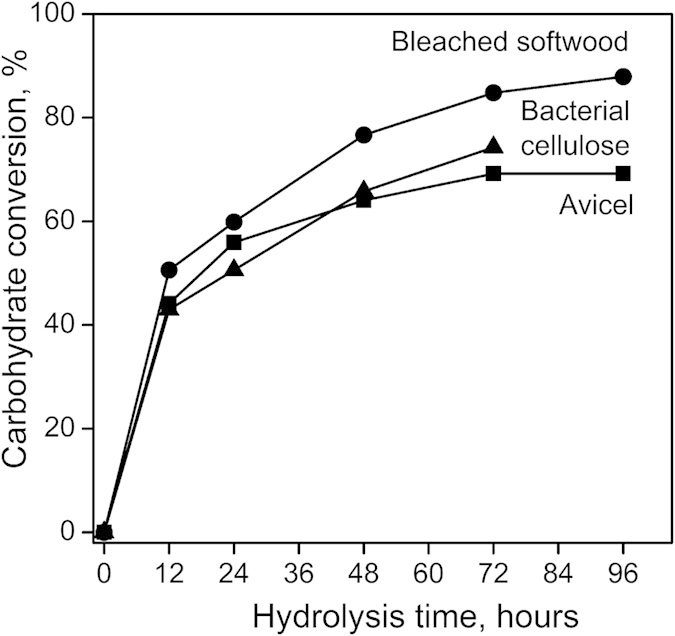
Enzymatic hydrolysis of the three cellulose substrates. Total carbohydrate conversion measured in Avicel (■), bleached softwood (∑), and bacterial cellulose (▲) over 96 hours. The hydrolysis of bacterial cellulose for 96 hours did not leave any solid residue.

Figure 1 shows the temporal profiles of total carbohydrate conversion yield during the enzymatic hydrolysis of Avicel, bleached softwood, and bacterial cellulose. The slope of each segment can be considered as an average conversion rate during the corresponding period. In all three cases, the conversion rate during the first 12-hour period was high, reaching a total conversion yield of about 44–50%. The conversion rate after 12 hours gradually decreased to a substantially lower value. Note that the temporal profiles shown in Fig. 1 cannot be explained by simple first-order reaction kinetics since the activity of solid substrates does not vary linearly with the concentration. The difference in carbohydrate conversion between bleached softwood and Avicel was about 18%, which could be attributed to the hemicellulose present in the softwood sample.

### Changes in cellulose chain length during enzymatic hydrolysis

To assess if the decrease in hydrolysis rate is related to cellulose digestion, the average DP of cellulose in the samples retrieved at various time points of the enzymatic hydrolysis process was measured^31^. The DP of a cellulose chain, which can vary after biomass pretreatment as well as with biomass type, has been considered a factor affecting relative activities of endoglucanase and exoglucanase because the abundance of binding sites for these enzymes can be a function of cellulose DP or chain length^33^.

**Figure 2:**
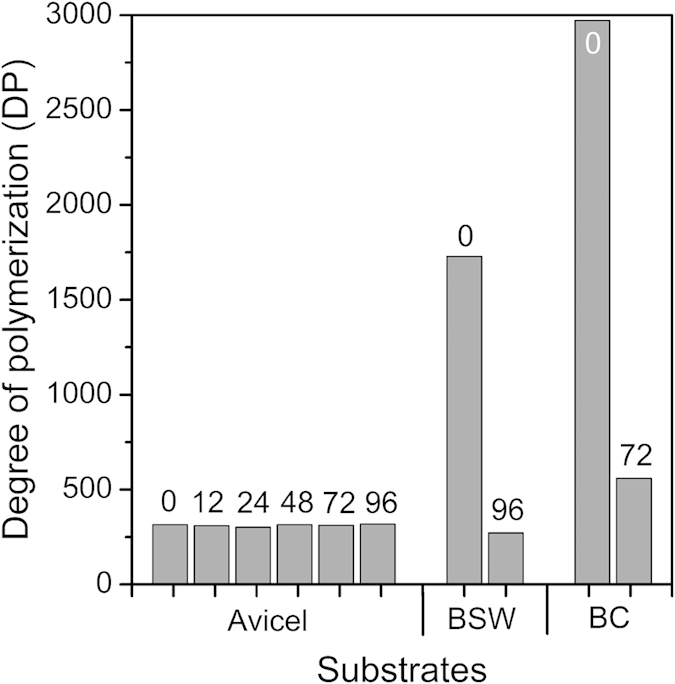
The weight average degree of polymerization (DP_w_) measured in the three cellulose substrates. DP values of Avicel, bleached softwood (BSW), and bacterial cellulose (BC) samples retrieved after enzymatic hydrolysis. The numbers on top of each bar plot represent the duration of enzymatic hydrolysis in hours.

In our study, the hydrolysis rate did not seem to correlate with changes in DP observed for all three cellulose substrates. As shown in Fig. 2, the DP of Avicel remained constant throughout the entire enzymatic hydrolysis process, although the enzymatic hydrolysis activity decreased over time. Avicel is crystalline powders produced through acid hydrolysis of pulps^34^; thus, its initial DP was low compared to the other samples. The constant DP for Avicel suggests that the enzyme can act only on the crystals exposed at the surface of the powders, while crystals deep inside the powder are not accessible by enzymes.

In the case of bleached softwood, the DP decreased substantially from ~1,700 to ~275. When enzymes penetrate into the bleached softwood cell walls through pores and preferentially consume amorphous cellulose and hemicellulose^4^,^6^, it could leave less reactive crystalline cellulose portion whose DPs are coincidentally the same as Avicel.

The DP of bacterial cellulose was initially ~3,000, which was the highest among the three samples analyzed in this study; it then reduced to ~560 after enzymatic hydrolysis. Bacteria synthesize cellulose in the absence of hemicellulose which could affect crystalline packing of cellulose chains^35^; thus, the DP of the cellulose in the indigestible portion of the bacterial cellulose might be different from those produced by plants. The inconsistency of the correlation between DP and hydrolysis activity for Avicel, bleached softwood, and bacterial cellulose implies that the DP of the cellulose substrate is not a key parameter causing the reduction of the hydrolysis rate of cellulose substrate over time.

### Cellulose crystallinity and crystal size estimated from XRD

The crystallinity of cellulose has been considered one of the primary factors that influence the rate of enzymatic hydrolysis^6^,^36^,^37^. Amorphous cellulose is known to be hydrolyzed faster than crystalline cellulose^4^,^6^. If the difference in the hydrolysis rate for amorphous versus crystalline phases is the main reason for the leveling-off trend in the conversion yield over time (Fig. 1), then one would expect that cellulose crystallinity should monotonically increase as the hydrolysis reaction continues. However, this hypothesis was not supported by XRD analyses in the current study.

**Figure 3:**
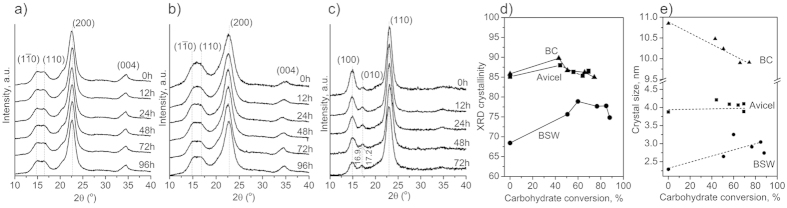
XRD diffractograms, crystallinity and crystal size analyses of the three cellulose substrates during the enzymatic hydrolysis. XRD diffractrograms of (**a**) Avicel, (**b**) bleached softwood (BSW), and (**c**) bacterial cellulose (BC) retrieved at different time points of enzymatic hydrolysis. (**d**) XRD crystallinity calculated using the peak-height method and (**e**) cellulose crystal size determined using the (200) diffraction peak for Avicel (■) and BSW (•) and the (110) peak for BC (▲).

Figure 3 shows the XRD analysis results for three biomass samples. The XRD crystallinity index was calculated using the peak height method for comparison purposes^17^. Determination of the cellulose crystallinity using XRD is method-dependent, and the values reported here should be taken as a qualitative measure only^38^. The crystallinity index values of Avicel and bacterial cellulose before hydrolysis were ~85% and initially showed a marginal increase; however, it decreased slightly after 40% conversion of total carbohydrate (*i.e*., after 12 hours of hydrolysis reaction) (Fig. 3d). The crystallinity index of bleached softwood showed much larger changes, increasing from ~67% before the reaction to ~80% after the first 12 hours of hydrolysis reaction (which corresponded to ~50% conversion of total carbohydrate); after 12 hours, the crystallinity index decreased slightly as the hydrolysis continued. The slight increase at the beginning could be interpreted as the preferential hydrolysis of amorphous cellulose over crystalline cellulose^5^,^39^. However, it could also be attributed to the fast hydrolysis of hemicellulose components present in the bleached softwood sample. It should be noted that the XRD crystallinity index is a rough estimate of the crystalline cellulose portion in the entire mass; it is not the value comparing crystalline versus amorphous cellulose portions^18^. The decrease of the crystallinity index after 12 hours could not be explained by the difference in hydrolysis rate of amorphous versus crystalline phases of cellulose in biomass. These results imply that the XRD crystallinity index change may not be the main cause for the decrease in enzymatic hydrolysis rate of these three biomass samples^40^.

One could hypothesize that the crystal size may affect the enzymatic hydrolysis activity since surface area varies with the crystal size. The crystal size estimation using the Scherrer equation (Fig. 3e) showed negligible changes for Avicel (~4 nm) throughout the hydrolysis period, a slight increase for bleached softwood (from 2.3 nm to ~3 nm after enzymatic hydrolysis), and a gradual decrease for bacterial cellulose (from 11 nm to 10.2 nm after 72 hours of reaction). Since the enzymatic hydrolysis will convert cellulose to soluble sugars, the crystal size increase of cellulose in bleached softwood cannot be the direct consequence of the hydrolysis reaction. This increase in crystal size must be due to side effects such as aggregation of cellulose microfibrils remaining in the cell walls after the digestion of hemicellulose between cellulose microfibrils or caused by the collapse of internal pores during the sample drying^11^,^12^.

On the other hand, the crystal size of bacterial cellulose decreased slightly with increasing hydrolysis duration. This could be interpreted as thinning of the cellulose microfibrils by hydrolysis^41^. However, the same was not observed for Avicel. Combining all these observations lead to a conclusion that the cellulose crystal size may not be the dominant factor causing the decreased hydrolysis rate. It could simply mean that XRD may not be sensitive enough to measure subtle changes in crystal size. In fact, it should be noted that the crystallinity estimation can be altered by the crystal size and vice versa 19,38.

### Changes in meso-scale packing of crystalline cellulose probed with SFG

The structural changes of cellulose microfibrils at the meso-scale (between ~10’s nm to a micron) could provide further information on how enzyme complexes and reaction products are transported inside the biomass^42^. The average size of pores in hydrated bleached softwood is larger than 20 nm^43^ while that of enzyme is about 5.9 nm^44^. The electron microscopy analysis of bacterial cellulose showed that pores between cellulose microfibrils are much larger than the size of enzymes, allowing penetration of enzymes through the entire sample^29^. Changes in these pore structures and dimensions during the enzymatic hydrolysis reaction could affect the reaction rate over time.

The surface morphology of biomass samples retrieved during enzymatic hydrolysis reactions has been investigated with atomic force microscopy (AFM), which reported opening of pores and fissures with increasing hydrolysis time^41^,^42^. However, AFM cannot show structural changes inside the biomass sample. In our previous studies, it was found that SFG could reveal various aspects of cellulose structures inside biomass samples^23^,^24^,^31^. When the volume fraction of crystalline cellulose, as well as the average size of cellulose crystals are relatively constant, then the spectral shapes and intensities of SFG can be correlated with the meso-scale packing of cellulose microfibrils^18^,^23^,^26^,^28^.

**Figure 4:**
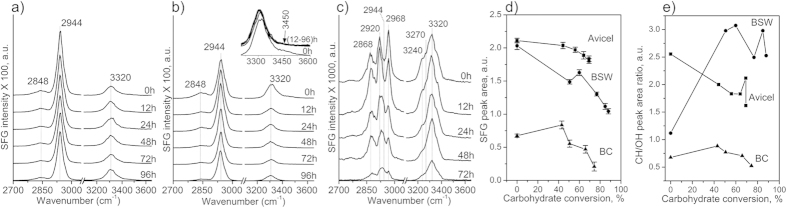
SFG spectra, overall peak intensity and peak area ratio of the three cellulose substrates during the enzymatic hydrolysis. SFG spectra of (**a**) Avicel, (**b**) bleached softwood (BSW), and (**c**) bacterial cellulose (BC) at different time points during the enzymatic hydrolysis. The OH region of BSW (normalized to 3,320 cm^−1^ peak) is shown in (**b**) inset to highlight the changes in 3,450 cm^−1^ shoulder peak in the first 12 hours of hydrolysis. (**d**) Changes in total SFG intensity (sum of peak area under CH_2_ and OH signals) and, (**e**) alkyl/hydroxyl SFG peak area ratio for three cellulose substrates as a function of total carbohydrate conversion yield. Error bars in d) is SEM (n = 10).

Figure 4 shows the SFG spectra of Avicel, bleached softwood, and bacterial cellulose retrieved after enzymatic hydrolysis at different times. The total SFG peak area showed only a marginal decrease with hydrolysis duration for Avicel (Fig. 4a). The bleached softwood sample showed a much larger decrease in SFG intensity as the hydrolysis yield increased (Fig. 4b). Specifically, it is noted that the OH SFG peak intensity at 3,320 cm^−1^ decreased drastically, and the shoulder peak at 3,450 cm^−1^ disappeared completely after the first 12 hours of enzymatic hydrolysis. The peak at 3,320 cm^−1^ is assigned to OH groups in the crystal interior^45^. The 3,450 cm^−1^ peak was speculated to originate from the surface hydroxyl groups of cellulose microfibrils, which can interact with hemicelluloses^24^,^46^. This shoulder peak is negligible in the SFG spectra of pure cellulose isolated from plant cell walls^23^. For example, the 3,450 cm^−1^ shoulder peak is absent in the SFG spectra of Avicel (Fig. 4a). Although SFG cannot directly detect amorphous hemicellulose, the disappearance of the 3,450 cm^−1^ peak (Fig. 4b inset) may suggest the fast hydrolysis of glucan chains close to the microfibril surfaces during the initial stage of enzymatic reactions. This supports the tentative assignment of this shoulder peak to the surface hydroxyl groups of cellulose microfibrils, which can participate in interactions with hemicellulose in plant cell walls.

The relative SFG intensity of the alkyl (CH and CH_2_) to hydroxyl (OH) peaks could be an indicator of cellulose meso-scale packing (Fig. 4e)^23^. The alkyl peak area is greater than the hydroxyl peak area for Avicel and bleach softwood (Fig. 4a,b), which is characteristic for antiparallel-packed cellulose microfibrils in secondary cell walls of land plants^23^. In bacterial cellulose, the alkyl and hydroxyl peak areas are comparable (Fig. 4c), which indicates randomly-arranged cellulose microfibrils^23^,^35^. These could be used as a basis set for interpretation of changes in the alkyl/hydroxyl SFG peak area ratio during enzymatic hydrolysis.

Avicel consists of random powders of short and densely-packed cellulose Iβ crystals^23^. This structure is not expected to change due to the enzymatic hydrolysis^40^. XRD analysis of Avicel showed that neither crystallinity nor crystal size varied substantially during the course of hydrolysis (Fig. 3a). Also, Avicel is nearly pure cellulose, thus the portion probed by SFG during hydrolysis is not expected to change. Knowing this, the slight decrease in alkyl/hydroxyl ratio indicates a reduced meso-scale packing in Avicel cellulose^40^. This apparent decrease may indicate a high degree of initial packing in the as-received commercial Avicel sample.

The bleached softwood is composed of very thin (a few nanometer diameters) and long microfibrils of cellulose Iβ embedded in a hemicellulose matrix. Both crystalline cellulose amount and apparent crystal size increased after enzymatic hydrolysis of bleached softwood (Fig. 3a). However, the overall SFG intensity of bleach softwood decreased gradually (Fig. 4d). It is noted that this decrease is mainly due to the decrease of the OH peak intensity. The increase in SFG alkyl/hydroxyl ratio could be ascribed to an increase in lateral packing of microfibrils^23^. The enhanced packing among cellulose microfibrils could result in a decrease in XRD peak width, which could be interpreted as an increase in the crystal size (Fig. 3e). The space between cellulose microfibrils created by enzymatic digestion could collapse into densely-packed structures during the drying process due to the surface tension of water^11^,^12^,^43^.

The overall SFG intensity of bacterial cellulose was lower than those of cellulose in Avicel and bleached softwood. This is mainly due to the difference in cellulose packing in these samples. Cellulose crystals in Avicel and microfibrils in woody cell walls are predominantly cellulose Iβ and laterally-packed in an antiparallel fashion^23^. In contrast, cellulose fibers in bacterial pellicles contain both Iα and Iβ allomorphs and are packed randomly^29^. The total SFG intensity of randomly-packed cellulose crystals is much weaker than that of laterally-packed crystals^23^. After 12 hours of hydrolysis with enzymes, the total SFG intensity of bacterial cellulose decreased significantly. Since neither XRD crystallinity nor crystal size varied substantially (Fig. 3d,e), the large decrease in SFG intensity must also be related to a decrease in meso-scale packing of cellulose microfibrils. Unlike the bleached softwood case, the alkyl/hydroxyl intensity ratio of bacterial cellulose did not change substantially (Fig. 4e). This might simply be because cellulose microfibrils in the bacterial sample remained random at all times.

### Adsorption of inactive species on cellulose substrate

The enzyme accessibility to cellulose substrate could be divided into two factors: (i) the transport of enzymes to cellulose surface through the matrix polymers in biomass and (ii) the availability of enzyme binding sites at the cellulose crystal or microfibril surfaces. The results shown in the previous section suggest that although the transport behavior of enzymes would be different for the three biomass samples studied here, all samples showed the same temporal behavior of hydrolysis rate. Ruling out the parameters considered so far, the remaining factor is the change in enzyme binding sites on the cellulose substrate over time. This hypothesis was tested by comparing the surface compositions of cellulose substrates before and after enzymatic hydrolysis reactions using XPS.

**Figure 5:**
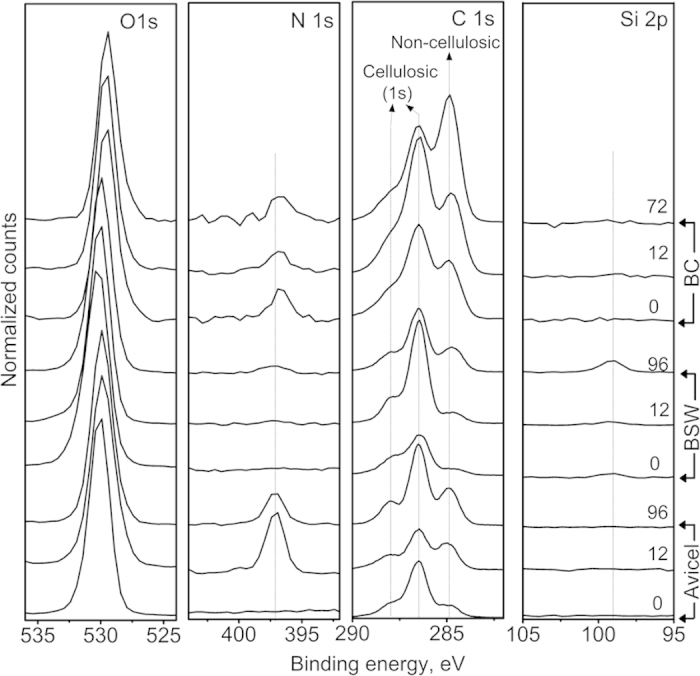
XPS analyses of the three cellulose substrates during the enzymatic hydrolysis. O1s, N1s, C1s, and Si2p XPS spectra of Avicel, bleached softwood (BSW), and bacterial cellulose (BC) before and after enzymatic hydrolysis reactions. The numbers in the Si2p panel represent the hydrolysis duration in hours. Each panel is offset in y-axis to avoid spectral overlap.

Figure 5 displays the high-resolution XPS spectra of C1s, O1s, N1s, and Si2p regions of three cellulose substrates. The chemical shift of the O1s peak is relatively small; thus, this peak was used to normalize the XPS peaks from different samples. The characteristic C1s peaks of cellulose are 286.7 eV and 288.1 eV (marked as ‘cellulosic’ in the C1s panel of Fig. 5)^47^,^48^. These peaks can be clearly seen in the XPS spectra of the 0 h samples of Avicel and bleached softwood. In the case of the 0 h sample of bacterial cellulose, there were significant N1s peaks (397 eV) and non-cellulosic C1s peaks (285 eV). These must be due to proteinaceous residues that could not be completely removed by the typical rinsing protocols for bacterial pellicles.

For all three substrates, XPS analysis results showed that the cellulose surface was covered by non-cellulosic substances after enzymatic hydrolysis reactions. In the case of Avicel, the nitrogen peak at 397 eV and the non-cellulosic component (285 eV) in the C1s region increased substantially, indicating the presence of enzymes. It was shown that solid substrates retrieved over the course of hydrolysis reactions are coated with proteinaceous species^49^,^50^. When those surface coating layers were removed by physical rinsing or proteinase, the cellulose hydrolysis rate was recovered^49^,^50^. These results suggested that the proteinaceous species on the Avicel surface detected in XPS must be denatured enzymes.

The nitrogen content in bleached softwood showed a small increase after the hydrolysis; at the same time, the Si2p peak increased noticeably. In plant cell walls, silica is deposited in intimate association with the organic components^51^. As cellulose and hemicellulose in bleached softwood are digested by enzymes, the silica particles could be accumulated at the biomass surface. In the case of bacterial cellulose, the large increase in the non-cellulosic components in the C1s spectrum was observed while the nitrogen content increase was not prominent. Again, this result is consistent with the deposition of non-cellulosic components, probably hydrolysis side products, on the cellulosic substrate.

Hence, among all parameters considered in this study, only the surface contamination revealed with XPS appears to be common for all three types of biomass and correlates well with the decrease in hydrolysis rate (Fig. 1). The adsorption of non-cellulosic species would evidently limit the enzyme binding sites on the cellulosic substrate, limiting the overall hydrolysis reaction yield. The changes in cellulose chain length (DP) were different for the three substrates. The XRD crystallinity and crystal size did not vary substantially for all three samples. SFG analysis results implied that the changes in meso-scale packing of cellulose crystals or microfibrils were different for all three substrates tested.

### Changes in cellulose polymorph content during enzymatic hydrolysis

The crystalline portion of bacterial cellulose contains 60–80% cellulose Iα, and the rest is cellulose Iβ^52^. The cellulase activity might be different for the two allomorphs of cellulose^53^. Due to subtle differences in their crystal structures, the distribution of surface binding sites for enzymes might differ for cellulose Iα and Iβ^54^. For algal biomass, it was reported that the cellulose Iα phase was digested faster than the cellulose Iβ phase^55^. A partial polymorphic change of cellulose III to cellulose I during enzymatic hydrolysis was also claimed previously^56^.

**Figure 6:**
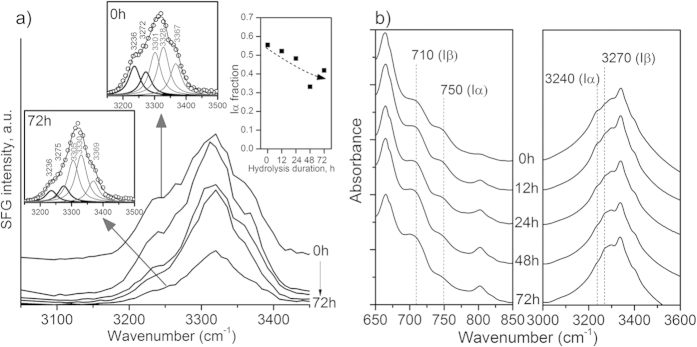
Changes in cellulose allomorph fraction in bacterial cellulose during the enzymatic hydrolysis. (**a**) SFG and (**b**) ATR-IR spectra of bacterial cellulose in the region sensitive to the cellulose polymorphic structure. The insets in (**a**) show the deconvolution of the OH stretch peaks as well as the Iα/(Iα + Iβ) ratio^23^.

In the current study, the change in cellulose allomorph fraction (Iα/Iβ ratio) in bacterial cellulose was evidenced from the SFG and ATR-IR analysis results (Fig. 6). In SFG, the OH peaks can be deconvoluted into at least five different components^23^,^35^. Among these components, the peaks at 3,240 cm^−1^ and 3,270 cm^−1^ are characteristic to cellulose Iα and Iβ, respectively. Thus, the ratio of these two components can be used to estimate the polymorphic ratio of cellulose. The deconvolution result showed that the Iα fraction had progressively decreased from 55% at 0 h to ~40% in 72 h sample (Fig. 6a inset). In ATR-IR spectra, two regions can be used to estimate the relative abundance of two polymorphs: one is the OH stretch region (same as SFG), and the other is the torsion or bending vibration of the glycosidic bond (<800 cm^−1^)^16^. The peak at 750 cm^−1^ belongs to cellulose Iα and the peak at 710 cm^−1^ to cellulose Iβ^21^. Figure 6b clearly shows that as the hydrolysis duration was increased, the cellulose Iα peaks at 750 cm^−1^ and 3,240 cm^−1^ became smaller compared to the cellulose Iβ peaks at 710 cm^−1^ and 3,270 cm^−1^.

In XRD (Fig. 3c), it was noted that the relative intensities of two peaks at 14° and 17° changed after enzymatic hydrolysis. These peaks correspond to the (100) and (010) planes for cellulose Iα and the 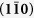 and (110) planes for cellulose Iβ^57^,^58^. Cellulose Iα usually shows a stronger peak at 14° compared to 17° (for example, see 0 h data in Fig. 3c), while two peaks are comparable to each other for cellulose Iβ (Fig. 3a,b)^35^,^57^. This is due to a difference in the cross-sectional shape of cellulose Iα and Iβ crystals^16^. Thus, the relative intensity change for these two peaks after 72 hours of enzymatic hydrolysis reaction is consistent with the decrease of the cellulose Iα component. In a closer look, the XRD peak position was also changed slightly: from 17.2° corresponding to the (010) value of cellulose Iα at 0 h to 16.9° corresponding to the (110) value of cellulose Iβ.

Combining all SFG, ATR-IR, and XRD analysis results of bacterial cellulose, it can be concluded that the cellulose Iα component is more susceptible to enzymatic hydrolysis than the cellulose Iβ component. Discussing the origin of this difference is beyond the scope of this work. In the literature, this difference for cellulose polymorphs has been attributed to variances in crystallographic facets of two allomorphs or the ‘core-shell’ structure of microfibrils containing both allomorphs^54^,^59^,^60^. Further studies are needed to elucidate the polymorphic differences in enzymatic hydrolysis activity for bacterial cellulose. However, unless cellulose Iα and Iβ samples with the identical crystallinity, crystal size, and meso-scale packing are prepared and used, the quantitative comparison of the absolute activity between these two allomorphs would be extremely difficult.

## Conclusions

To better understand the factors affecting the decrease in enzymatic hydrolysis activity over time, a number of structural characterizations were performed. The accessibility of the cellulose microfibril surface was found to be a common factor for the tested substrates: Avicel, bleached softwood, and bacterial cellulose. The XRD crystallinity, crystal size, degree of polymerization, and changes in meso-scale packing did not directly correlate with temporal changes in enzymatic hydrolysis. In addition, the allomorphic change in bacterial cellulose over the course of enzyme hydrolysis suggested a preferential hydrolysis of cellulose Iα over cellulose Iβ.

## Additional Information

**How to cite this article**: Kafle, K. *et al.* Progressive structural changes of Avicel, bleached softwood, and bacterial cellulose during enzymatic hydrolysis. *Sci. Rep.* **5**, 15102; doi: 10.1038/srep15102 (2015).

## Figures and Tables

**Table 1:** Composition analysis of the Avicel and Bleached softwood substrate.

	Glc%	Xyl%	Ara, Man%	Total carbo%	KL%	ASL%	Lignin%	Mass Balance%
Avicel	97.6	—	—	97.6	—	—	—	97.6
BSW	73.9	9.5	7.5	90.9	—	0.6	0.6	91.5

KL = Klason lignin; ASL = Alcohol soluble lignin

## References

[b1] Lynd L. R., Cushman J. H., Nichols R. J. & Wyman C. E. Fuel ethanol from cellulosic biomass. Science 251, 1318–1323 (1991). 17816186 10.1126/science.251.4999.1318

[b2] Zhang Y. H. P. & Lynd L. R. Toward an aggregated understanding of enzymatic hydrolysis of cellulose: noncomplexed cellulase systems. Biotechnol. Bioeng. 88, 797–824 (2004). 15538721 10.1002/bit.20282

[b3] Mansfield S. D., Mooney C. & Saddler J. N. Substrate and enzyme characteristics that limit cellulose hydrolysis. Biotechnol. Progr. 15, 804–816 (1999). 10.1021/bp9900864 10514250

[b4] Sasaki T., Tanaka T., Nanbu N., Sato Y. & Kainuma K. Correlation between X‐ray diffraction measurements of cellulose crystalline structure and the susceptibility to microbial cellulase. Biotechnol. Bioeng. 21, 1031–1042 (1979).

[b5] Sinitsyn A., Gusakov A. & Vlasenko E. Y. Effect of structural and physico-chemical features of cellulosic substrates on the efficiency of enzymatic hydrolysis. Appl. Biochem. Biotechnol. 30, 43–59 (1991).

[b6] Fan L. T., Lee Y.-H. & Beardmore, D. Major chemical and physical features of cellulosic materials as substrates for enzymatic hydrolysis. Adv. Biochem. Eng. 14, 101–117 (1980).

[b7] Ramos L., Nazhad M. & Saddler J. Effect of enzymatic hydrolysis on the morphology and fine structure of pretreated cellulosic residues. Enzyme Microb. Technol. 15, 821–831 (1993).

[b8] Puri V. P. Effect of crystallinity and degree of polymerization of cellulose on enzymatic saccharification. Biotechnol. Bioeng. 26, 1219–1222 (1984). 18551639 10.1002/bit.260261010

[b9] Thompson D. N., Chen H.-C. & Grethlein H. E. Comparison of pretreatment methods on the basis of available surface area. Bioresour. Technol. 39, 155–163 (1992).

[b10] Grethlein H. E. Pretreatment for enhanced hydrolysis of cellulosic biomass. Biotechnol. Adv. 2, 43–62 (1984). 14543719 10.1016/0734-9750(84)90240-4

[b11] Langan P. *et al.* Common processes drive the thermochemical pretreatment of lignocellulosic biomass. Green Chem. 16, 63–68 (2014).

[b12] Kafle K. *et al.* Effects of delignification on crystalline cellulose in lignocellulose biomass characterized by vibrational sum frequency generation spectroscopy and x-ray diffraction. Bioenergy Res. 10.1007/s12155-015-9627-9 (2015).

[b13] Nishiyama Y., Langan P., O’Neill H., Pingali S. & Harton S. Structural coarsening of aspen wood by hydrothermal pretreatment monitored by small- and wide-angle scattering of X-rays and neutrons on oriented specimens. Cellulose 21, 1015–1024 (2014).

[b14] Jeoh T. *et al.* Cellulase digestibility of pretreated biomass is limited by cellulose accessibility. Biotechnol. Bioeng. 98, 112–122 (2007). 17335064 10.1002/bit.21408

[b15] Wan J., Wang Y. & Xiao Q. Effects of hemicellulose removal on cellulose fiber structure and recycling characteristics of eucalyptus pulp. Bioresour. Technol. 101, 4577–4583 (2010). 20181478 10.1016/j.biortech.2010.01.026

[b16] Kim S. H., Lee C. M. & Kafle K. Characterization of crystalline cellulose in biomass: Basic principles, applications, and limitations of XRD, NMR, IR, Raman, and SFG. Korean J. Chem. Eng. 30 2127–2141 (2013).

[b17] Park S., Baker J. O., Himmel M. E., Parilla P. A. & Johnson D. K. Cellulose crystallinity index: measurement techniques and their impact on interpreting cellulase performance. Biotechnol. Biofuels 3, 1–10 (2010). 20497524 10.1186/1754-6834-3-10 PMC2890632

[b18] Barnette A. L. *et al.* Quantification of crystalline cellulose in lignocellulosic biomass using sum frequency generation (SFG) vibration spectroscopy and comparison with other analytical methods. Carbohydr. Polym. 89, 802–809 (2012). 24750865 10.1016/j.carbpol.2012.04.014

[b19] French A. D. & Cintrón M. S. Cellulose polymorphy, crystallite size, and the Segal Crystallinity Index. Cellulose 20, 583–588 (2013).

[b20] Lee C. M. *et al.* Cellulose polymorphism study with sum-frequency-generation (SFG) vibration spectroscopy: identification of exocyclic CH_2_OH conformation and chain orientation. Cellulose 20, 991–1000 (2013).

[b21] Imai T. & Sugiyama J. Nanodomains of Iα and Iβ Cellulose in Algal Microfibrils. Macromolecules 31, 6275–6279 (1998).

[b22] Gama F. & Mota M. Enzymatic hydrolysis of cellulose (II): X-ray photoelectron spectroscopy studies on cellulase adsorption. Effect of the surfactant Tween 85. Biocatal. Biotransform. 15, 237–250 (1997).

[b23] Lee C. M., Kafle K., Park Y. B. & Kim S. H. Probing crystal structure and mesoscale assembly of cellulose microfibrils in plant cell walls, tunicate tests, and bacterial films using vibrational sum frequency generation (SFG) spectroscopy. PCCP 16, 10844–10853 (2014). 24760365 10.1039/c4cp00515e

[b24] Park Y. B. *et al.* Monitoring meso-scale ordering of cellulose in intact plant cell walls using sum frequency generation spectroscopy. Plant Physiol. 163, 907–913 (2013). 23995148 10.1104/pp.113.225235 PMC3793067

[b25] Barnette A. L. *et al.* Selective detection of crystalline cellulose in plant cell walls with sum-frequency-generation (SFG) vibration spectroscopy. Biomacromolecules 12, 2434–2439 (2011). 21615075 10.1021/bm200518n

[b26] Kafle K. *et al.* Cellulose microfibril orientation in onion (*Allium cepa* L.) epidermis studied by atomic force microscopy (AFM) and vibrational sum frequency generation (SFG) spectroscopy. Cellulose 21, 1075–1086 (2014).

[b27] Kafle K., Greeson K., Lee C. & Kim S. H. Cellulose polymorphs and physical properties of cotton fabrics processed with commercial textile mills for mercerization and liquid ammonia treatments. Text. Res. J. 84, 1692–1699 (2014).

[b28] Kafle K. *et al.* Vibrational sum-frequency-generation (SFG) spectroscopy study of the structural assembly of cellulose microfibrils in reaction woods. Cellulose 21, 2219–2231 (2014). 10.1039/c4cp00515e 24760365

[b29] Lee C. M., Gu J., Kafle K., Catchmark J. & Kim S. H. Cellulose produced by Gluconacetobacter xylinus strains ATCC 53524 and ATCC 23768: Pellicle formation, post-synthesis aggregation and fiber density. Carbohydr. Polym. 133, 270–276 (2015). 26344281 10.1016/j.carbpol.2015.06.091

[b30] Sluiter A. *et al.* Determination of structural carbohydrates and lignin in biomass *Laboratory Analytical Procedures (LAP), National Renewable Energy Laboratory (NREL)* (2008) Available at: http://www.nrel.gov/biomass/analytical_procedures.html Accessed: July 2012.

[b31] Wang W. *et al.* Effect of mechanical disruption on the effectiveness of three reactors used for dilute acid pretreatment of corn stover Part 1: chemical and physical substrate analysis. Biotechnol. Biofuels 7, 57 (2014). 24713111 10.1186/1754-6834-7-57 PMC3999883

[b32] Segal L., Creely J., Martin A. & Conrad C. An empirical method for estimating the degree of crystallinity of native cellulose using the X-ray diffractometer. Text. Res. J. 29, 786–794 (1959).

[b33] Hallac B. B. & Ragauskas A. J. Analyzing cellulose degree of polymerization and its relevancy to cellulosic ethanol. Biofuels, Bioprod. Biorefin. 5, 215–225 (2011).

[b34] Rojas J., Lopez A., Guisao S. & Ortiz C. Evaluation of several microcrystalline celluloses obtained from agricultural by-products. J. Adv. Pharm. Technol. Res. 2, 144–150 (2011). 22171310 10.4103/2231-4040.85527 PMC3217711

[b35] Park Y. B. *et al.* Effects of plant cell wall matrix polysaccharides on bacterial cellulose structure studied with vibrational sum frequency generation spectroscopy and x-ray diffraction. Biomacromolecules 15, 2718–2724 (2014). 24846814 10.1021/bm500567v

[b36] Cao Y. & Tan H. Study on crystal structures of enzyme-hydrolyzed cellulosic materials by X-ray diffraction. Enzyme Microb. Technol. 36, 314–317 (2005).

[b37] Hall M., Bansal P., Lee J. H., Realff M. J. & Bommarius A. S. Cellulose crystallinity–a key predictor of the enzymatic hydrolysis rate. FEBS J. 277, 1571–1582 (2010). 20148968 10.1111/j.1742-4658.2010.07585.x

[b38] Lee C. M. *et al.* Correlations of apparent cellulose crystallinity determined by XRD, NMR, IR, Raman, and SFG methods. Adv. Polym. Sci. 10.1007/12_2015_320 (2015).

[b39] Gao S., You C., Renneckar S., Bao J. & Zhang Y.-H. P. New insights into enzymatic hydrolysis of heterogeneous cellulose by using carbohydrate-binding module 3 containing GFP and carbohydrate-binding module 17 containing CFP. Biotechnol. Biofuels 7, 1–11 (2014). 24552554 10.1186/1754-6834-7-24 PMC3943381

[b40] Penttilä P. A. *et al.* Changes in submicrometer structure of enzymatically hydrolyzed microcrystalline cellulose. Biomacromolecules 11, 1111–1117 (2010). 20329744 10.1021/bm1001119

[b41] Santa-Maria M. & Jeoh T. Molecular-scale investigations of cellulose microstructure during enzymatic hydrolysis. Biomacromolecules 11, 2000–2007 (2010). 20583829 10.1021/bm100366h

[b42] Bubner P., Plank H. & Nidetzky B. Visualizing cellulase activity. Biotechnol. Bioeng. 110, 1529–1549 (2013). 23456755 10.1002/bit.24884

[b43] Park S., Venditti R. A., Jameel H. & Pawlak J. J. Changes in pore size distribution during the drying of cellulose fibers as measured by differential scanning calorimetry. Carbohydr. Polym. 66, 97–103 (2006).

[b44] Tanaka M., Ikesaka M., Matsuno R. & Converse A. O. Effect of pore size in substrate and diffusion of enzyme on hydrolysis of cellulosic materials with cellulases. Biotechnol. Bioeng. 32, 698–706 (1988). 18587771 10.1002/bit.260320515

[b45] Lee C. M., Mohamed N. M. A., Watts H. D., Kubicki J. D. & Kim S. H. Sum-frequency-generation vibration spectroscopy and density functional theory calculations with dispersion corrections (DFT-D2) for cellulose I*α* and I*β*. J. Phys. Chem. B 117, 6681–6692 (2013). 23738844 10.1021/jp402998s

[b46] Fernandes A. N. *et al.* Nanostructure of cellulose microfibrils in spruce wood. PNAS 108, E1195–E1203 (2011). 22065760 10.1073/pnas.1108942108 PMC3223458

[b47] Johansson L. S. & Campbell J. Reproducible XPS on biopolymers: cellulose studies. Surf. Interface Anal. 36, 1018–1022 (2004).

[b48] Popescu C.-M., Tibirna C.-M. & Vasile C. XPS characterization of naturally aged wood. Appl. Surf. Sci. 256, 1355–1360 (2009).

[b49] Yang B., Willies D. M. & Wyman C. E. Changes in the enzymatic hydrolysis rate of Avicel cellulose with conversion. Biotechnol. Bioeng. 94, 1122–1128 (2006). 16732604 10.1002/bit.20942

[b50] Yu Z., Jameel H., Chang H.-m., Philips R. & Park S. Evaluation of the factors affecting avicel reactivity using multi-stage enzymatic hydrolysis. Biotechnol. Bioeng. 109, 1131–1139 (2012). 22125215 10.1002/bit.24386

[b51] Jones L. Mineral components of plant cell walls. Am. J. Clin. Nutr. 31, S94–S98 (1978). 707400 10.1093/ajcn/31.10.S94

[b52] Watanabe K., Tabuchi M., Morinaga Y. & Yoshinaga F. Structural features and properties of bacterial cellulose produced in agitated culture. Cellulose 5, 187–200 (1998).

[b53] Mittal A., Katahira R., Himmel M. E. & Johnson D. K. Effects of alkaline or liquid-ammonia treatment on crystalline cellulose: changes in crystalline structure and effects on enzymatic digestibility. Biotechnol. Biofuels 4, 1–16 (2011). 22011342 10.1186/1754-6834-4-41 PMC3219654

[b54] Beckham G. T. *et al.* Molecular-level origins of biomass recalcitrance: decrystallization free energies for four common cellulose polymorphs. J. Phys. Chem. B 115, 4118–4127 (2011). 21425804 10.1021/jp1106394

[b55] Hayashi N., Sugiyama J., Okano T. & Ishihara M. The enzymatic susceptibility of cellulose microfibrils of the algal-bacterial type and the cotton-ramie type. Carbohydr. Res. 305, 261–269 (1997).

[b56] Ciolacu D., Gorgieva S., Tampu D. & Kokol V. Enzymatic hydrolysis of different allomorphic forms of microcrystalline cellulose. Cellulose 18, 1527–1541 (2011).

[b57] Wada M., Okano T. & Sugiyama J. Allomorphs of native crystalline cellulose I evaluated by two equatoriald-spacings. J. Wood Sci. 47, 124–128 (2001).

[b58] French A. Idealized powder diffraction patterns for cellulose polymorphs. Cellulose 21, 885–896 (2014).

[b59] Yamamoto H., Horii F. & Hirai A. *In situ* crystallization of bacterial cellulose II. Influences of different polymeric additives on the formation of celluloses I_α_ and I_β_ at the early stage of incubation. Cellulose 3, 229–242 (1996).

[b60] Horikawa Y., Clair B. & Sugiyama J. Varietal difference in cellulose microfibril dimensions observed by infrared spectroscopy. Cellulose 16, 1–8 (2009).

